# CLensRimVision: A Novel Computer Vision Algorithm for Detecting Rim Defects in Contact Lenses

**DOI:** 10.3390/s23239610

**Published:** 2023-12-04

**Authors:** Pawat Chunhachatrachai, Chyi-Yeu Lin

**Affiliations:** 1 Department of Mechanical Engineering, National Taiwan University of Science and Technology, Taipei 10632, Taiwan; pawat.chun@gmail.com; 2 Center for Intelligent Manufacturing Innovation, National Taiwan University of Science and Technology, Taipei 10632, Taiwan

**Keywords:** contact lens, computer vision, automatic optical inspection, defect detection, image processing

## Abstract

Automated optical inspection (AOI) plays a pivotal role in the quality control of contact lenses, safeguarding the safety and integrity of lenses intended for both medical and cosmetic applications. As the role of computer vision in defect detection expands, our study probes its effectiveness relative to traditional methods, particularly concerning subtle and irregular defects on the lens rim. In this research study, we propose a novel algorithm designed for the precise and automated detection of rim defects in contact lenses called “CLensRimVision”. This algorithm integrates a series of procedures, including image preprocessing, circle detection for identifying lens rims, polar coordinate transformation, setting defect criteria and their subsequent detection, and, finally, visualization. The method based on these criteria can be adapted either to thickness-based or area-based approaches, suiting various characteristics of the contact lens. This approach achieves an exemplary performance with a 0.937 AP score. Our results offer a richer understanding of defect detection strategies, guiding manufacturers and researchers towards optimal techniques for ensuring quality in the contact lens domain.

## 1. Introduction

Contact lenses, medical devices intended to be worn on the cornea, have revolutionized the world of ophthalmology and vision correction [1,2]. Their use has grown substantially over the years due to their convenience and advancements in materials and design [3,4,5]. Ensuring the safety and effectiveness of these lenses is crucial, not only to provide optimal vision but also to prevent potential eye-related complications [6,7]. One of the critical aspects for ensuring the quality of contact lenses is the detection and elimination of defects, particularly in areas that have a direct interface with the eye [8,9,10].

The rim area of a contact lens is particularly sensitive as defects in this region can lead to discomfort, tear film disruption, or even potential injury to the ocular surface. Traditional methods for defect detection, such as manual inspection, may not provide the necessary sensitivity or specificity needed for accurate and efficient quality control at an optimal cost and in an optimal time frame [11,12]. The size of a contact lens is very tiny relative to the human body. It must be in a proper liquid, comfortable, visible, transparent, soft, and curved. These are challenges for the quality control process of the product, which has strict criteria for its use in humans.

In recent times, with the advent of the computer, there has been a paradigm shift in how inspections are conducted in various industries [13,14,15,16,17,18]. Automated defect detection systems have demonstrated high levels of accuracy and consistency, surpassing human capabilities in certain cases. However, the unique challenges posed by the transparent nature of contact lenses and the subtle tiny defects that can arise in the rim area require a specialized approach.

**Figure 1:**
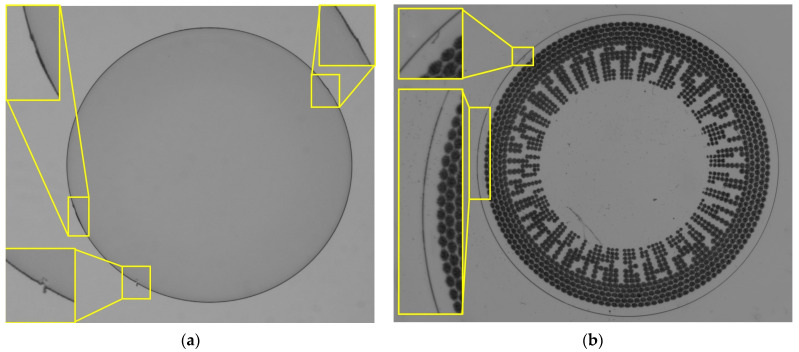
Sample defects on edge of contact lens. (**a**) Clear contact lens; (**b**) Colored and patterned contact lens.

This paper introduces a novel algorithm tailored for the robust and accurate detection of defects in the rim area of contact lenses. Leveraging advanced image processing techniques combined with mathematics and statistics, this system aims to offer superior detection capabilities, ensuring higher quality and safety standards for contact lens users worldwide. We focus on this area because it directly interacts with the rim of the human eye, making it crucial for the wearer’s comfort. Defects, such as burrs, can cause irritation to wearers. Figure 1a,b illustrate examples of a clear lens and a colored and patterned contact lens edge with some defects and a lack of smoothness from our real dataset. This kind of defect is called a burr, which refers to a small, unwanted, and often sharp-edged or -ridged material that can form on the surface of a workpiece as a result of various machining or manufacturing processes. In the yellow box, display a larger view of samples showing defects in the rim area of both transparent and patterned contact lenses.

Our paper’s contributions are as follows.

- We proposed a novel image-processing-based algorithm to detect burr defects on the rim of contact lenses.
- We applied this comprehensively using mathematics and statistics to a computer vision algorithm.
- We presented information relevant for manufacturers and researchers in the contact lens sector, providing them with insights and methodologies that can be directly applied to improve quality control automation processes.

## 2. Background

Contact lenses are intricately designed medical devices that require the utmost precision during manufacturing. Defects, even if minute, not only compromise the wearer’s visual clarity but also their safety. Over the years, ensuring defect-free lenses has been a priority, with multiple quality control methods put into place.

Historically, the contact lens industry has relied heavily on manual inspection methods, in which trained professionals visually inspect each lens under magnification. While this method has the advantage of human intuition, it is time-consuming and susceptible to inconsistencies and errors, especially when dealing with vast production numbers.

### 2.1. Quality Control in Optical Industry

In the realm of optical products, such as contact lenses and camera lens glasses, maintaining a high level of quality control is paramount to ensuring optimal transparency [7,8,9] and visual performance, as Rebsamen et al. stated [19]. While defects in optical products can occur, quality control systems are in place to assess whether the detected defects meet the acceptable criteria. It is important to note that different areas within the same product may have distinct criteria for evaluation [20].

For contact lenses specifically, quality control standards are outlined in ISO 18369-1:2017 and contact lens fundamental requirements in ISO 14534:2015 [19]. These standards not only address the overall quality control requirements but also delve into the criteria and responsibilities for the different areas of a contact lens. Adhering to these standards ensures that the quality of contact lenses aligns with established guidelines and contributes to the overall reliability and safety of these optical products. The most inner circle zone is called the center optical zone, Zone 2 is called the rim optical zone, and Zone 1 is called the rim connected zone. In our research, we will solely focus on Zone 1 as it is the area most sensitive to the user’s safety and the area we use to develop our algorithm for the detection of edge defects. 

### 2.2. Computer Vision for AOI of Contact Lenses

AOI is being used to shorten the process of the mass production of lenses in terms of minimizing the presence of defective products. There are several techniques and combinations inside the AOI sector. In the context of contact lens defect detection, Chang et al. use computer vision algorithms to utilize edge points to calculate the distance from the center and employ a radius abnormality function [21]. This approach involves computing the distance between the radii of adjacent parts of the circle and evaluating the absolute difference. Finally, the radius abnormality function is applied by defining an offset threshold. However, during the actual experimental setup, there may be situations in which the contact lens is not properly placed in the contact lens holder (we will explain this in Section 3.1), leading to an uneven thickness along the circular rim of the contact lens (we will discuss this in Section 4.4.1). This scenario motivates us to further develop the algorithm to accurately detect defects even under such circumstances, a consideration that Cheng et al. may not have considered.

After scoping the contact lens area, the next step is to use image processing methods to effectively detect and assess defects in contact lenses, which can contribute to overall quality control. The utilization of computer vision algorithms enhances the efficiency and accuracy of the AOI process for contact lens production. The following image preprocessing and computer vision techniques make the defect clearer and more accurately detected.

#### Image Processing Techniques

- Color space and morphological operations can extract information as compared to intensity or HSV color-space-based segmentation, as Dumitru Dan Burdescu et al. proved in their research [22]. The HSV (hue, saturation, and value) color space is utilized for color-based object segmentation. The hue channel represents the color category, while the saturation ranges from unsaturated to fully saturated levels. The value channel conveys the color intensity. Morphological operations, such as erosion and dilation, modify images using a structuring element [23,24,25]. Hasan et al. use morphological operations and prove that this technique can detect the edge of objects [26]. Moreover, the usage of erosion reduces noise and isolates elements, while dilation merges dissimilar elements and detects irregularities.
- Polar Coordinate Transformation: The conversion of Cartesian coordinates (x, y) to polar coordinates (r, θ) has various effects and facilitates specific image processing tasks. This transformation is useful for radial distortion correction, circular pattern analyses, and the transformation of images into a polar representation. The open library from CV2 [27] that supports this algorithm is Warp polar [28].
- Circle Detection: The algorithm aims to identify the best-fit circle that matches a given set of points. A circle in 3D space can be represented using a parametric equation, involving the center, radius, normal unit vector, and perpendicular vector. These parameters provide a comprehensive description of the circle in three-dimensional space. Ramlee et al. use circle detection to detect outliers in their research [29,30], which is similar to our aim to detect burr defects on the circular edge of contact lens.

## 3. Experimental Setup

### 3.1. Concept of Machine Design

**Figure 2:**
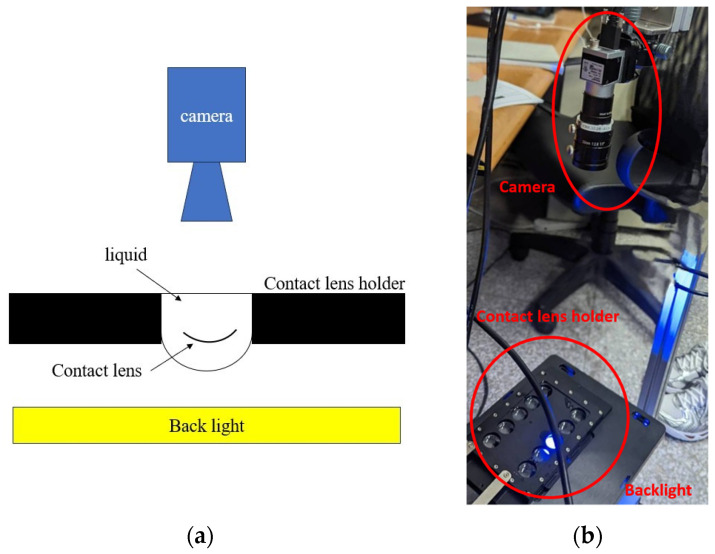
Design of contact lens AOI system. (**a**) Setup design; (**b**) Physical setup.

This section outlines the procedural steps for configuring the traditional physical design of a contact lens. The automated optical inspection (AOI) system is designed to capture high-resolution images of contact lenses and emphasize detailed visualization of defects. The setup design is shown in Figure 2a. First, to set up the camera, position it facing downward from the top of the inspection holder. Second, set up the base, which contains the contact lens in a liquid. The container must be transparent and placed beneath the camera. Third, since contact lenses are transparent objects, a specialized photographic approach employing backlighting is crucial. A backlight is placed at the bottom of the inspection base to illuminate the object of interest and reveal all the necessary details for the camera to capture. In our study, we adopted a traditional approach inspired by the design of this contact lens AOI machine. Our camera placement was carefully calibrated to enable surface imaging, while backlighting was used to detect irregularities or foreign substances. The design of this apparatus draws from the work of Jin et al. [31,32], who used cold cathode fluorescent lamps (CCFLs) as a light source to illuminate a glass ribbon placed perpendicular to the vertical axis. The image was then captured using a CCD camera. The physical arrangement in our experiment is presented in Figure 2b. This equipment was designed and built by Intelligent Robotics Lab, Department of Mechanical Engineering, National Taiwan University of Science and Technology.

After setting up the station and successfully capturing images of an adequate quality for the experiment, the next step involves performing the experiment on a processing unit. For this experiment, we utilize a computer with the following specifications: an Intel^®^ Core™ i9-10900X CPU running at 3.70 GHz, an RTX 1080Ti 11 GB GPU, and 16 GB of RAM.

### 3.2. Dataset 

**Figure 3:**
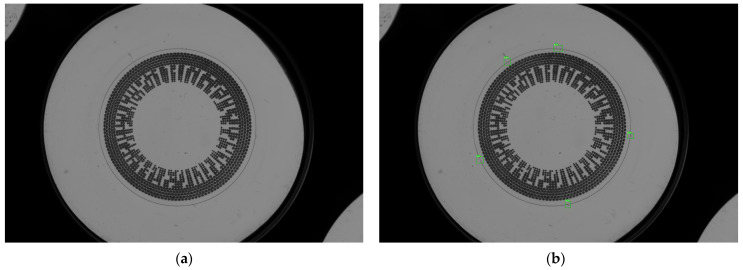
A sample of contact lens. (**a**) Original image; (**b**) Labeled image.

This dataset serves as the foundation for testing the novel algorithm using image processing approaches. Our objective is to assess the inherent learning capabilities of algorithm using raw images alongside evaluation of their corresponding labels. The raw images we use for training and evaluation are 5472 pixels in width and 3648 pixels in height. The original set of contact lens samples comprised 60 lenses; 3 images for each were captured, producing a total of 180 original images as shown in Figure 3.

The dataset was composed of two types of contact lenses: a clear contact lens and colored and patterned contact lens, which are shown in Figure 1a,b, respectively. Transparent contact lens has 12 pieces and colored and patterned contact lens has 48 pieces in the dataset. 

We prepared the dataset for evaluation metrics by labeling the defect area of each image. The labeled defects are shown in Figure 3. In each image, the defects are labelled in details in the green boxes. The objective is to decrease high variation in detection results’ aspect ratio and size. Examples of the original field of view (FOV) and an image with the visualized labels are shown in Figure 3a,b, respectively. 

### 3.3. Evaluation Metrics 

In this section, we introduce key evaluation metrics that play a pivotal role in assessing the performance of our object detection model. These metrics provide valuable insights into the accuracy and completeness of object localization and identification, which are fundamental aspects of our research.

- Intersection over union (IoU) is a critical metric in object detection, quantifying the degree of overlap between predicted bounding boxes and ground truth bounding boxes. It is calculated as the ratio of the area of intersection between the two bounding boxes to the area of their union. IoU serves as a measure of the spatial alignment between the predicted and actual object locations.
- Precision measures the proportion of true positive predictions out of all positive predictions made by the model. In our context, it assesses how many of the objects our model detected are genuinely correct.
- Recall measures the proportion of true positive predictions out of all actual objects in the dataset. It evaluates our model’s capability to identify all relevant objects in the images.
- Mean average precision (mAP) is a comprehensive metric that combines precision and recall across a range of IoU thresholds. It provides a single scalar value to gauge the overall performance of our object detection model. mAP is particularly valuable when we need to compare our model’s performance against various benchmarks or evaluate its effectiveness across different scenarios. In the context of our research, the dataset exclusively comprises a single class of objects. Consequently, we have opted to utilize average precision (AP) as the primary evaluation metric. AP offers a robust means of assessing the precision/recall trade-off and model performance and is particularly well suited for single-class object detection scenarios.

In the subsequent sections, we will delve into the methodology of our experiments, the datasets used, and the results obtained, all of which are pivotal in demonstrating the effectiveness of our approach.

## 4. Methodology

**Figure 4:**
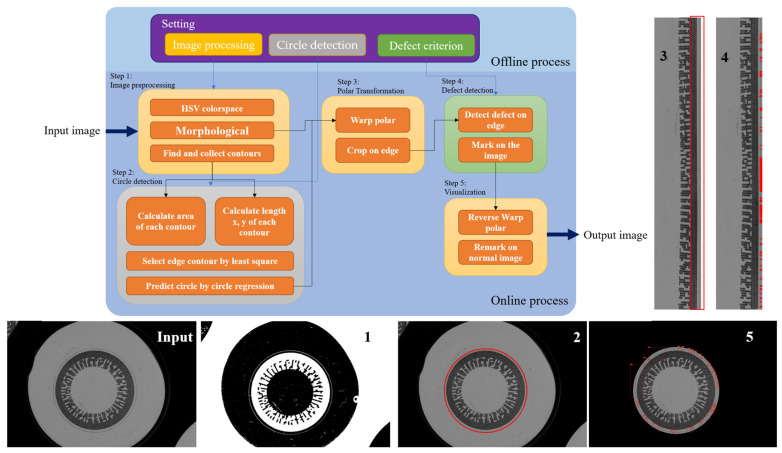
ClenRimVision algorithm process.

This chapter explains the concept of CLensRimVision algorithm to detect defects on the edge of the outer circle of the contact lens. ClensRimVision algorithm comprises two main components: the offline process and the online process. The term ‘offline process’ refers to the setting of parameters which must be fully established prior to implementation or production. We use ‘offline’ to emphasize that these preparations are essential and must be completed beforehand. Conversely, the ‘online process’ pertains to the actual implementation of the algorithm, specifically for defect detection on a production line. The workflow of ClensRimVision algorithm is as shown in Figure 4. In this proposed algorithm, we integrate both basic image processing methods and our designed methods within the overall workflow. The description of basic image processing is provided in Section 4.1, Section 4.2, Section 4.3, Section 4.4 and Section 4.5. However, the sequence for connecting each method is determined and arranged by us. This arrangement has been proven to guarantee the desired output results and performance of our experiments. The innovative part that can significantly increase our chance to fulfill our objective, as explained in Section 4.4, constitutes the core idea of this research paper. The sequencing of the system workflow is also a significant aspect contributing to the innovation of this research. Each step in the workflow plays a crucial role in enhancing the results of the preceding part, as it serves as the input for the subsequent stage.

In the offline process, various parameters are carefully configured to optimize the performance of the defect detection algorithm. During implementation, images of the contact lens may be captured under different settings, such as various methods of capture, lighting conditions, distances, and more. Therefore, proposing an algorithm that can dynamically adjust parameters according to different settings is essential. This entails setting parameters for three key aspects: image preprocessing, circle detection, and defect detection, as shown in Table 1. These parameters are predetermined and serve as the foundation for the defect detection system. In the online process, the algorithm utilizes the configured parameters to analyze and detect defects on the edges of contact lenses. This is achieved through the application of image processing procedures and the proposed algorithms. The online process takes input images or a combination of computer vision modules as input and performs defect detection based on the configured parameters from the offline process.

Furthermore, the algorithm’s workflow comprises five main execution steps as follows:

1. Image Preprocessing: This step is responsible for adjusting and preprocessing the input contact lens image to convert it into a contour image, highlighting the rich details and essential components within the image. In Figure 4, labelled “Input” shows the input image of ClensRimVision algorithm, and labelled “1” shows the output of the Image Preprocessing step.
2. Circle Detection: This step focuses on detecting the area and coordinates of the circular contact lens within the input image. The red circle in Figure 4, labelled “2” shows output of Circle Detection step.
3. Polar Coordinate Transformation: This step focuses on the transformation from circular to linear forms, stemming from the circle detection step. In Figure 4, labelled “3” shows image after Polar Coordinate Transformation is applied, and in the red box shows the region of interest for the algorithm.
4. Defect Detection: This step focuses on detecting defects at the rim area by using linear image. In Figure 4, labelled “4” shows defects in the object by printing red area in the image.
5. Visualization: This step focuses on visualizing the defects by reversing polar coordinate transformation and marking the results. In Figure 4, labelled “5” shows result of detecting defects in the original image aspect.

### 4.1. Image Preprocessing 

We employ image processing to refine and enhance the input image, making edges sharper and more readily detectable by the circle algorithm. This enhancement utilizes two primary functions in image processing: firstly, the HSV color space, and secondly, the morphology method.

A color space is a multidimensional system by which colors can be represented, usually within three or more dimensions. Each dimension corresponds to a distinct color component. Recognizing the differences between various color spaces is pivotal in image processing, as the chosen color space can profoundly influence tasks such as segmentation, representation, and image manipulation.

Morphological image processing focuses on the shape or morphology of features within an image. Rooted in set theory, this method provides tools for extracting components of an image that are instrumental in describing and representing region shapes, like boundaries, skeletons, and the convex hull. The primary applications of morphological processing include noise reduction and sealing the contour of the circular edge of contact lenses using two core operations: dilation and erosion.

**Figure 5:**
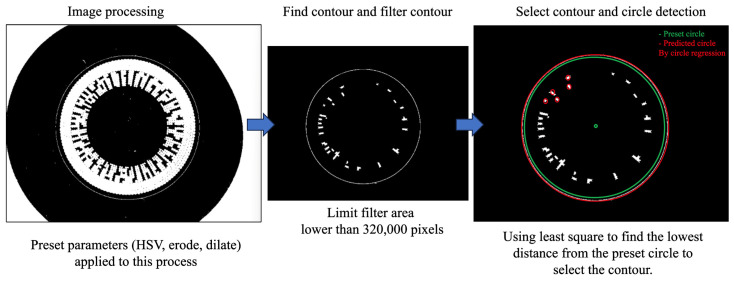
The workflow of image processing and circle detection steps.

Thus, our image preprocessing involves using the HSV color space to filter and pinpoint the section within our desired color range as in Figure 5, which, in this context, is the rim of a contact lens. Subsequently, morphological image processing is employed to minimize noise and seal the circular rim of the contact lens. Following this preprocessing step, the algorithm identifies the image contours and progresses to the subsequent phase: circle detection. The final image then undergoes the image processing polar coordinate transformation. 

### 4.2. Circle Detection 

In this study, we opted for circle regression due to its efficiency and robustness to noise, enabling us to efficiently detect circular objects in complex, cluttered images without the need for extensive parameter tuning, thereby streamlining the image analysis process [27,33]. In this section, the input of this step is the list of contours from the first step. The algorithm needs to pick the rim contour by first filtering the size of the contour lower than parameter named “Max area of contour” which can reduce many contours from noise and container part; secondly, by selecting the final contour by using the least squares equation compared to parameters including “size of radius circle in range” and “center circle position”, as shown in Figure 5; and then finally, by obtaining a very accurate circle parameter to be able to continue to the next part, which is the polar coordinate transformation. We use circle regression to fit the points from the contours. Circle fitting refers to the problem of finding a circle that best fits a set of data points with the least squares method. This method can be employed in a variety of scientific and engineering contexts, such as computer vision, robotics, and metrology.

Given a set of data points ($x_{i}$, $y_{i}$), the general equation of a circle is as follows:

(1) $${\left(x_{i}-a\right)}^{2}+{\left(y_{i}-b\right)}^{2}=r^{2}$$

where (a, b) are the coordinates of the circle’s center and r is the radius of the circle.

For the circle fitting, the goal is to determine the values of a, b, and r that minimize the sum of the squared distances or error between the data points and predicted circle. In this paper, we used linearization method to solve the circle fitting by starting with the standard circle question in Equation (2).

(2) $$x^{2}+{\;y}^{2}+\mathrm{Dx}+\mathrm{Ey}+F=0$$

with

(3) D=−2a

(4) E=−2b

(5) $$F={\;a}^{2}+b^{2}-r^{2}$$

Solve Equations (2)–(5) to find a, b, and r and perform a least squares linear regression to fit the equation. 

### 4.3. Image Processing for Polar Coordinates’ Transformation

The purpose of this section is to transform the circle image to a linear image. The polar coordinate transformation discussed in this section applies different geometrical modifications to 2D images. While they do not alter the actual content of the image, they reshape the pixel grid and project this reshaped grid onto the output image. To ensure the best quality and avoid any distortion, the projection is carried out in the opposite direction: from the output back to the input. This means that for every pixel (x, y) in the output image, the functions determine the respective original pixel in the input image and transfer its value. The linear image can apply one-dimensional window sliding, with the optimal computation cost of the algorithm for calculating information about the edges of a contact lens, such as area and thickness.

### 4.4. Defect Detection 

**Figure 6:**
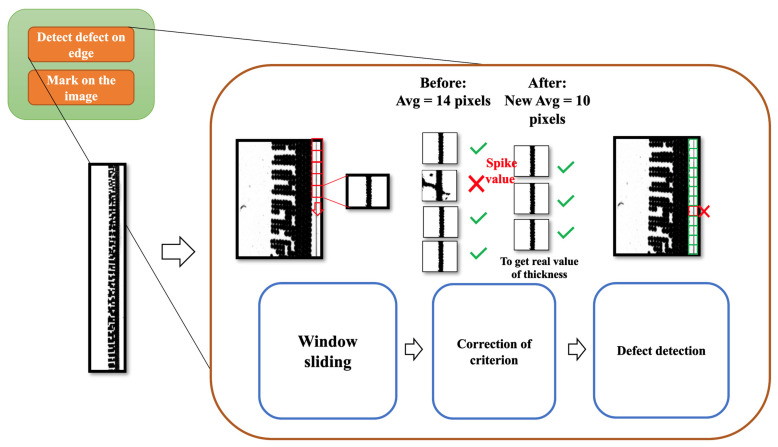
The workflow of defect detection.

The defect detection step is composed of three substeps, including contour calculation using window sliding, defining the correction criteria, and finding an acceptable range for calculation. The flowchart of defect detection is shown in Figure 6.

The overall concept of defect detection in the algorithm is that it slices the long image into small pieces. Then, it selects a range of interested numbers of windows to calculate the average and standard deviation (SD). After obtaining the average and SD, the equation is used to determine if the value in the previously calculated window meets the condition. If so, then it is gathered for the second round of average value calculation. Lastly, the new average value is used for defect detection using the criteria for the acceptable range.

#### 4.4.1. Contour Calculation Using Window Sliding

**Figure 7:**
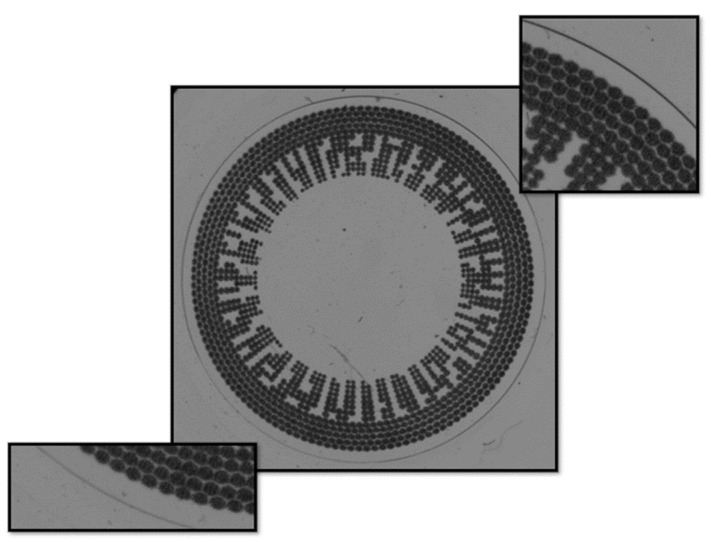
Edge of a contact lens with uneven thickness.

Window sliding solves the problem of the edge line of the contact lens does not have an uneven thickness along the whole circle, as shown in Figure 7. In this scenario, if we create an algorithm to obtain the data from the whole circle and calculate the statistical result, it would be biased, and unfortunately, half of the thicker circle may be detected as a defect. 

**Figure 8:**
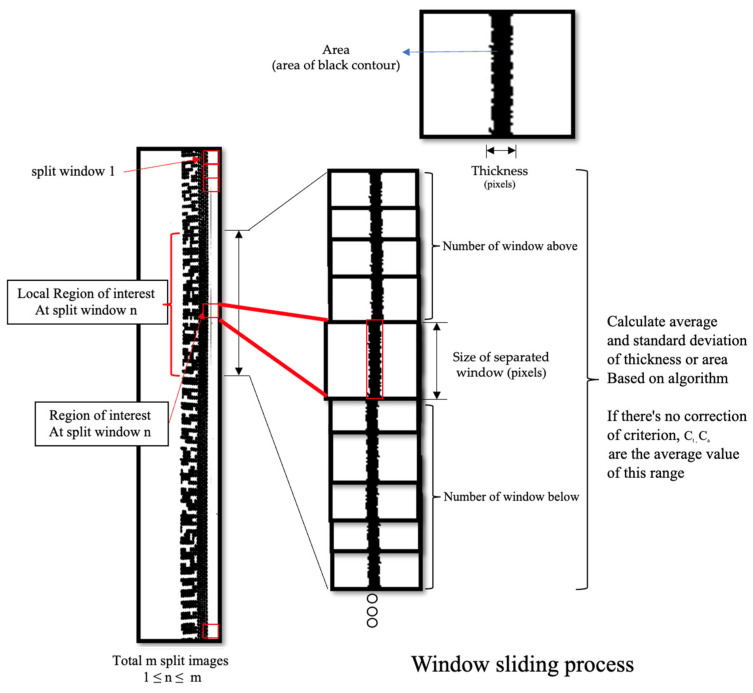
The sliding window process and an explanation of the parameters.

To avoid that, we perform the window-sliding technique to set a range of windows and obtain the data from the local area where it has a similar ground truth thickness. First, the algorithm starts with performing window sliding, at which point it receives the window size parameter as input along with the straightened preprocessed image. The size will be set to the window vertical height of all windows in the input image. The demonstration of window sliding in our algorithm is as shown in Figure 8.

Second, we select the method to count black contours. As you can see in Figure 8, the black contours along the image are uneven with some rough edges. The red box represents a window section for the window sliding method. To determine whether these surfaces are defects, we proposed two approaches to perform the calculation, which are area-based approach and thickness-based approach. 

For the area-based approach, the algorithm counts the number of black contours in pixels inside each window using OpenCV library. For a thickness-based approach, the algorithm counts the maximum width of black contour along the *x*-axis. This can be achieved by using OpenCV library.

The reason for proposing two different approaches is to compare their performance and check either of these approaches would be suitable for different objectives of defect detection. For example, let us assume that the average thickness in the example of 10 × 10 window slides is four with SD of one and the average number of black pixels (area) is 35 with SD of 10. If we consider only thickness of the black contour, there could be a case in which the thickness seems normal but somehow the defect is a slight swelling. In this case, area-based approach would detect and count the number of black pixels and return an unusually high value of black pixels inside that window. Another possibility is that, if a thin spike occurs in an image, the area-based approach may perform calculations and find that this window’s black pixel value is normal; however, if we used thickness-based approach, it would immediately know that there is a spike in this window. 

After calculating the value of black contours in each window, either using area-based approach or thickness-based approach, the last step is to calculate the average and SD values from a selected range of window slides. In this part, the parameter “number of windows above and below” is used to decide the range of each calculation. The reason we calculated the average is to use it as a determined value for the later step. To provide a clear explanation of each step, we present the procedure of this step in pseudocode in Algorithm 1.
**Algorithm 1:** Defect Criteria–Window-sliding method**Input**   (1) Preprocessed image  (2) Size_window = parameter “Size of separated image”  (3) Num_above = parameter “Number of images above”  (4) Num_below = parameter “ Number of images below”**Output**  (1) Criteria_based_list = the list contain in the local region of interest range either “thickness_based_list” or “area_based_list”.  (2) Criteria_avg = the average of Criteria list (“Criteria_list_value”)  (3) Criteria_sd = the standard deviation of Criteria list (“Criteria_list_value”)**Begin**  1. Image_total_length = Preprocessed_image.length  2. Number_of_window = Image_total_length/size_window  3. Criteria_based_list = []  4. **If** (area_based is True) then  5.  **For** (window = 0, window=Number_of_window, window=window+size_window) then  6.     Criteria_based_list = Area_based_list.append(find_area_of_contour(i))  7. **else** then  8.  **For** (window=0, window=Number_of_window, window=window+size_window) then  9.     Criteria_based_list = Thickness_based_list.append(find_thickness_of_contour(i))  10. //find average  11. Window_AVG_list, Window_SD_list = [], []  12. **For** (window=0, window= Criteria_based_list.length, window=window+1) then  13.  Window_local_range = Criteria_based_list[window − Num_above : window + Num_below]  14.  Window_local_AVG = Window_AVG_list.append(Average[window_local_range])  15.  Window_local_SD = Window_SD_list.append(SD[window_local_range])  16.  //correction of criterion (will be explained in the next section)  17.  //defect detection (will be explained in the next section)**End**

#### 4.4.2. Correction of Criteria

This substep is proposed to slightly adjust the average from the previous substep to be more accurate as the thickness and area of the contact lens rims. The calculated average value from the previous step may include the value of the defects, which may lead to an unusually high value of the average. To detect this, we proposed the correction criteria method to reevaluate the average value by excluding the outliers in consideration. The first step for correction criteria is to check each window’s black contour value with the set criteria that uses the average and SD values from the previous step. The equation for correction criteria is as shown in Equation (6).

Average − F × SD ≤ qualified value ≤ Average + F × SD (6)

**Figure 9:**
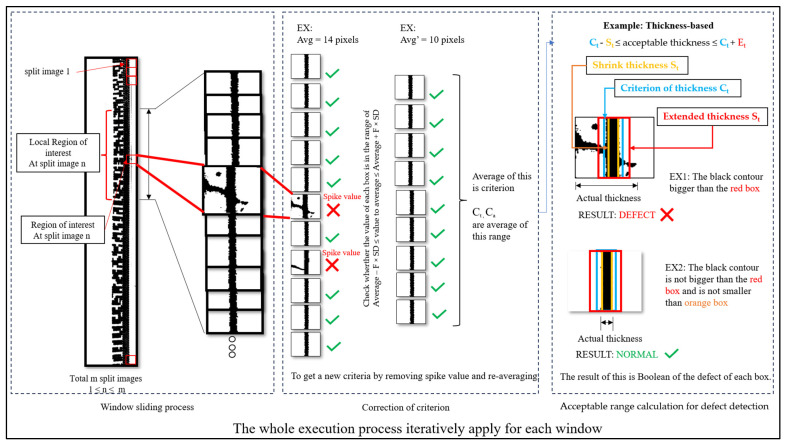
The execution process in a window sliding calculation.

The average and SD values are specified from each set of windows for each iteration. The factor of SD to average is the required input parameter. If the window’s black contour value is within the range of lower bound and upper bound, then that window’s value is gathered and used for calculating a new average value. Figure 9 demonstrates how valid windows are selected for the calculation of a new average. 

The pseudocode used for demonstrating the concept in this step is as shown in Algorithm 2.
**Algorithm 2:** Defect Criteria–Correction of Criterion**Input**  (1) Window_local_range = either Thickness_based_list or Area_based_list  (2) Factor_of_SD as parameter “Factor of SD to average”  (3) AVG as Window_local_AVG  (4) SD as Window_local_SD**Output**  (1) Window_new_local_AVG**Begin**  1. //find new average in correction of criterion, the for loop(line3) is the same in last loop in Algorithm 1  2. **For** (window = 0, window= Criteria_based_list.length, window=window+1) then//line 12 in Algorithm 1  3.  //window sliding return AVG, SD, Window_local_range  4.   Window_new_local_range= []  5.   **For** (window=0, window= Window_local_range.length, window=window+1) then  6.      **If** (AVG − (SD*Factor_of_SD) < Criteria_based_list[window] < AVG + (SD*Factor_of_SD))  7.        Window_new_local_range = Window_new_local_range.append(Criteria_based_list [window])  8.   Window_new_AVG = Average [Window_new_local_range]  9.   **Return** Window_new_AVG**End**

#### 4.4.3. Calculation of Acceptable Range for Defect Detection

The last step in defect detection on the circle edge of the contact lens is to perform the defect detection. In this step, the algorithm is prompted with equations that determine whether the window has a burr defect. The equations are separately designed for area-based approach and thickness-based approach, in which the lower bound is multiplied by the shrink value, and the upper bound is multiplied by extended value. These two values have different domains for area-based and thickness-based approaches. For a thickness-based approach, it is calculated with the instant value of pixel as shown in Equation (7). For area-based approach, the extended and shrink values are calculated with the percentage of the average of the black contour area as shown in Equation (8).

C_t_ − S_t_ ≤ acceptable thickness ≤ C_t_ + E_t_ (7)

C_a_ − S_a_ ≤ acceptable area ≤ C_a_+ E_a_ (8)

From Equations (7) and (8), C represents the new average value after being calculated from the correction criteria algorithm. S represents the shrink value and E represents the extended value. For the area-based approach, the subscription symbol a is used to represent this method, while the symbol t represents the thickness-based approach. With this equation, if any window’s value has the value outside the equation range, it is considered a defect. Algorithm 3 shows the pseudocode for the calculation of the acceptable range based on 4 inputs, including the output of Algorithm 2, the shrink criterion, the extended criterion and the window value.

In this stage of the process, the algorithm undertakes a couple of significant actions. Initially, it overlays a red mask onto the image to enhance visualization and to help in assessing how well the algorithm is performing. Simultaneously, it employs a specific image processing technique, which we will delve into in the next section. In terms of performance assessment, the algorithm relies on these enhanced images to calculate evaluation metrics. This assessment involves comparing the algorithm’s results to a labeled dataset, as we will discuss in detail in the following sections focusing on evaluation metrics and dataset information.
**Algorithm 3:** Acceptable range calculation for defect detection**Input**  (1) New_AVG as Window_new_local_AVG or Window_local_AVG incase using with or without correction of criterion function respectively.  (2) Shrink_criterion is a parameter from either “Shrink thickness” or “Shrink area”.  (3) Extended_criterion is a parameter from wither “Extended thickness” or “Extended area”.  (4) Window_value is a thickness or area value of a certain window.**Output**  (1) List Boolean of the defect as Bools_results**Begin**  1. //defect detection  2. Bools_results = []  3. **For** (window=0, window= Criteria_based_list.length, window=window+1) then//line 12 in Algorithm 1  4.  //window sliding: return AVG, SD, Criteria_based_list  5.  //correction of criterion: return Window_new_local_AVG  6.  //start defect detection  7.   New_AVG = Window_new_local_AVG//shorten the variable name  8.   **If** (New_AVG − Shrink_criterion < Criteria_based_list[window] < New_AVG + Extended_criterion)  9.      Bools_results.append(True)  10.   **Else** Bools_results.append(False)  11. **Return** Bools_results**End**

### 4.5. Image Processing for Visualization

**Figure 10:**
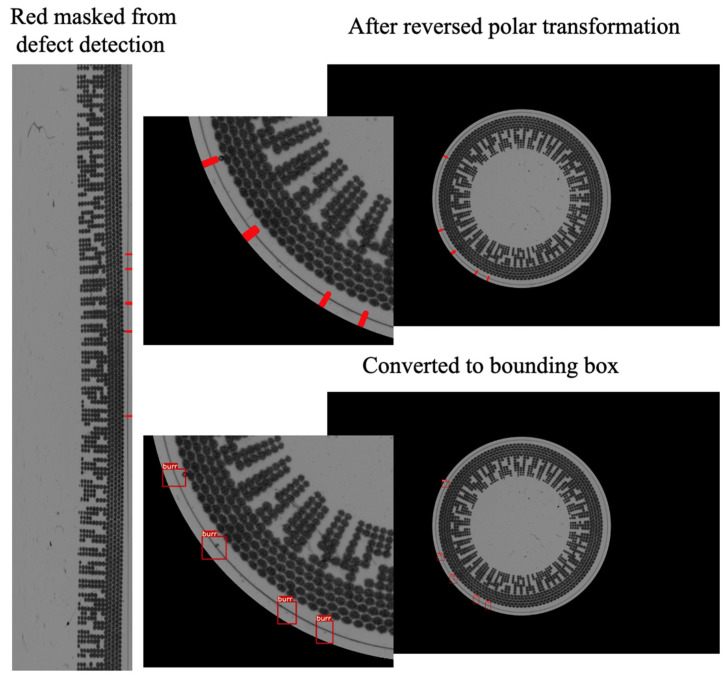
Image processing for visualization result.

In this step, the reverse function of the polar coordinate transformation is employed. The predicted circle coordinates (x, y) and radius (r) obtained from the circle detection are used as input for this reverse function. The polar transformation equation is a reversible function used to convert the linear image back into a circular image, as illustrated in Figure 10. In the process of defect detection, the algorithm will mask the red contour on the defect following the window size in which the contour represents a rectangle size in the image before reversing polar transformation.

In the output from the defect detection step, the image region containing defects is highlighted in red. The algorithm utilizes a color space method for image processing to extract the red contours, convert them into bounding areas, and present the result, as depicted in Figure 10.

## 5. Results

In the previous section, we proposed a novel algorithm that can perform defect detection at the rim area of contact lens bycalculating criteria by either thickness-based or area-based. In this section, we present the results of our algorithm’s performance for different configurations and criteria. Table 2 summarizes the outcomes for both the “thickness-based” and “area-based” approaches. These results demonstrate the influence of varying shrink and extended thickness criteria, the number of images above and below a specified pixel size of split images, and the factor correction applied to the criteria. The values of the parameter that we set in each trial of the experiment were chosen intentionally. We carefully selected the values for each parameter through a series of experimental trials. For the shrink/extended criteria, we opted for values that were closely aligned with the original, introducing only slight variations to explore the optimal parameter settings that would be the most effective acceptable range to detect defects in the contact lens. When it came to the “Number of windows above/below” and “Factor correction of criterion”, both were set as fixed parameters. The “Number of windows above” was established as 20 because this configuration involves 20 windows above and 20 windows below the window currently focused on, totaling 40 windows. This exceeds the threshold of 30, as stipulated by the central limit theorem [34]. The “Factor correction of criterion” defaults to 0.5 to maintain proximity to the average value and mitigate excessive fluctuations. Regarding the “Pixel size of split images”, we employed values of 10 and 60. This choice was informed by our preliminary study of burr defects, which indicated a statistical range spanning from 8 to 52 pixels for each burr’s position. Given the considerable variability in the burr sizes, we set the pixel size of the split images to 60 to encompass the largest burrs. However, we also assessed the detection performance for smaller burrs using a smaller split image size of 10. By examining the results for these two pixel sizes, we can discern meaningful contrasts and draw further conclusions from the data.

### 5.1. Thickness-Based Approach

**Figure 11:**
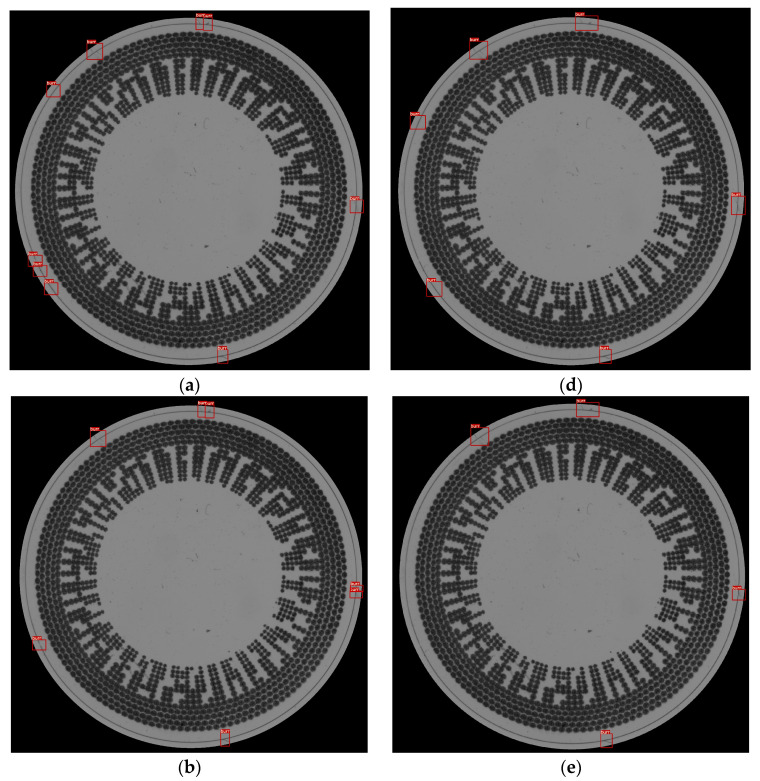

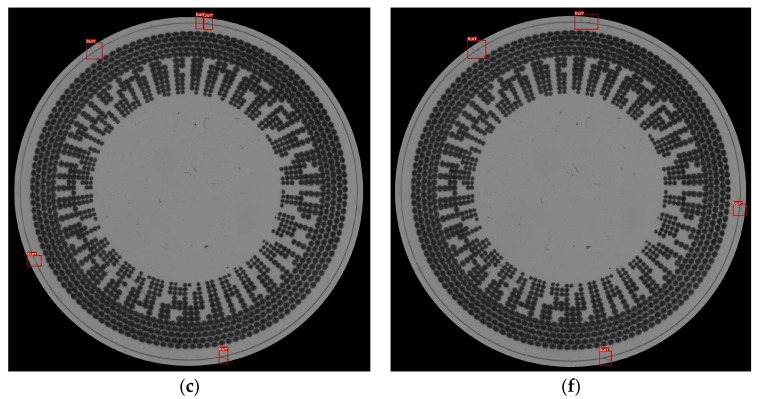
Results from a sample using the thickness-based method with different settings. (**a**) 10-pixel image splitting combined with a 6-pixel extended and shrink thickness criterion; (**b**) 10-pixel image splitting combined with an 8-pixel extended and shrink thickness criterion; (**c**) 10-pixel image splitting combined with a 10-pixel extended and shrink thickness criterion; (**d**) 60-pixel image splitting combined with a 6-pixel extended thickness criterion; (**e**) 60-pixel image splitting combined with an 8-pixel extended and shrink thickness criterion; and (**f**) 60-pixel image splitting combined with a 10-pixel extended and shrink thickness criterion.

The “thickness-based” algorithm was evaluated using three different shrink/extended thicknesses (6, 8, and 10) and two distinct pixel sizes of the split images (10 and 60). The results indicated that for the 10-pixel split images, the algorithm achieved a high precision of 0.950 and 0.911 for the thicknesses of 6 and 10, respectively. However, as the pixel size increased to 60, the precision remained high but slightly decreased. This trend showcases the algorithm’s ability to adapt to varying image characteristics while maintaining a good balance between precision and recall. Figure 11 shows an example of the result of different split image sizes; for the pixel heights of 10 and 60, the thicknesses are between 6 and 10 in 2-unit increments (6, 8, and 10 pixels). So, there are six image results for this sample. The contact lens from the examples below has inconsistent edges. The algorithm can detect but cannot cover all of the inconsistent edges. The example below represents the results of detecting burr defects on a contact lens.

In Figure 11, the 10-pixel split image performs better for the shrink defect criteria in the detection of the bottom part; the predicted results are shown for the thicknesses of 6, 8, and 10. In another setting, the 60-pixel split image cannot detect the same defect. Thus, the 10-pixels split image cannot detect the extended edge defect at the right side of the contact lens with a thickness of 10 pixels. However, the 60-pixel split image can perform well under all of the criteria settings.

### 5.2. Area-Based Approach

For the “area-based” algorithm, we examined different factor correction values (0.3, 0.4, and 0.5) and two different pixel sizes for split images (10 and 60). Notably, the algorithm consistently demonstrated a robust performance with precision values above 0.9 for various factor correction values. This suggests that the “area-based” method maintains its efficacy across different configurations. 

**Figure 12:**
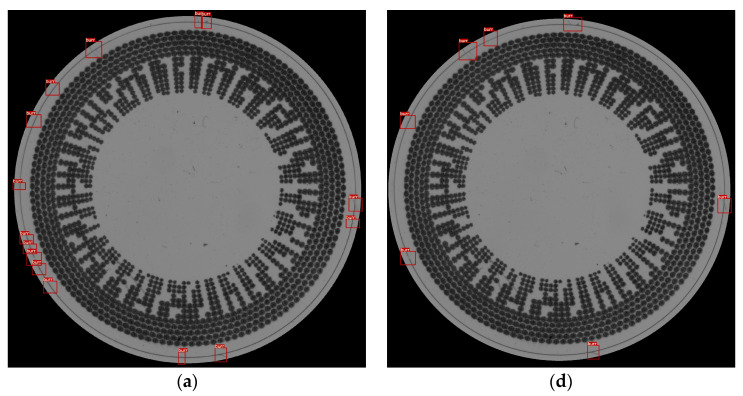

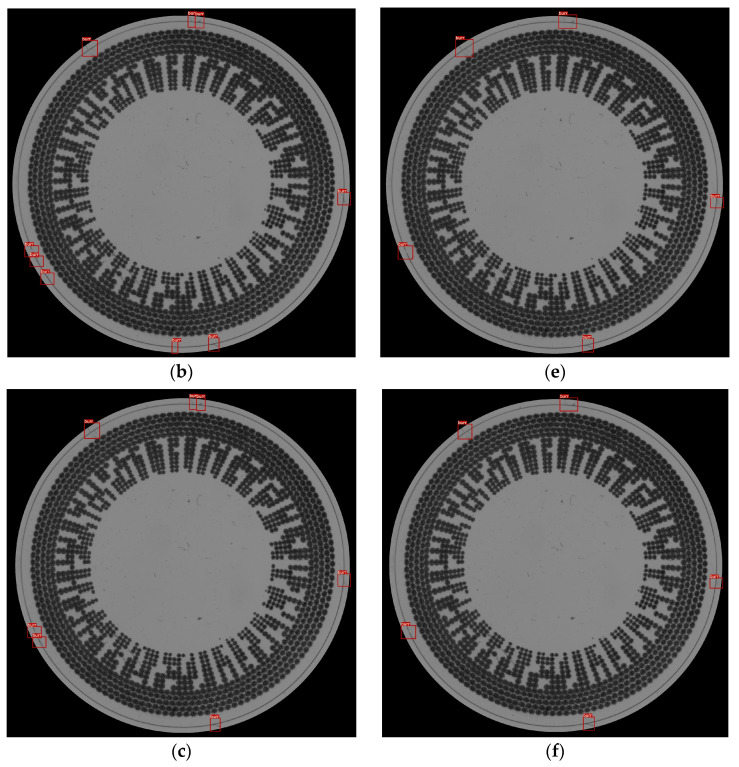
Results from a sample using the area-based method with different settings. (**a**) 10-pixel image splitting combined with threshold of 0.3 extended and shrink area criterion; (**b**) 10-pixel image splitting combined with threshold of 0.4 extended and shrink area criterion; (**c**) 10-pixel image splitting combined with threshold of 0.5 extended and shrink area criterion; (**d**) 60-pixel image splitting combined with threshold of 0.3 extended and shrink area criterion; (**e**) 60-pixel image splitting combined with threshold of 0.4 extended and shrink area criterion; (**f**) 60-pixel image splitting combined with threshold of 0.5 extended and shrink area criterion.

Figure 12 shows a sample of the results of the area-based approach with the same samples as the thickness-based approach. This method also can adjust the parameters for the change criterion and split image size. In this method, the split image size is not set as a high value because this will affect the percentage of the area of the criteria for detection. Therefore, this parameter value is set to 10 pixels and 60 pixels, while the percentage of the area in the criteria is set as three values: 0.3, 0.4, and 0.5.

The lower split image size of 10 pixels has more prediction detection bounding boxes. It shows that the sensitivity of the model of the setting of the smaller split image is higher, as shown in the left part of the result in Figure 12a–c. Also, the top part of the result is split into two bounding boxes, but Figure 12d–f has the one bigger box in the top part of the predicted result. In the bigger size of the split image, the number of predicted bounding boxes is obviously lower than that of the smaller split image size. This shows that the precision of the setting in which the 60-pixel size is used is greater than that of the 10-pixel split image size. 

## 6. Discussion

### 6.1. Effectiveness of Window-Sliding Method

**Figure 13:**
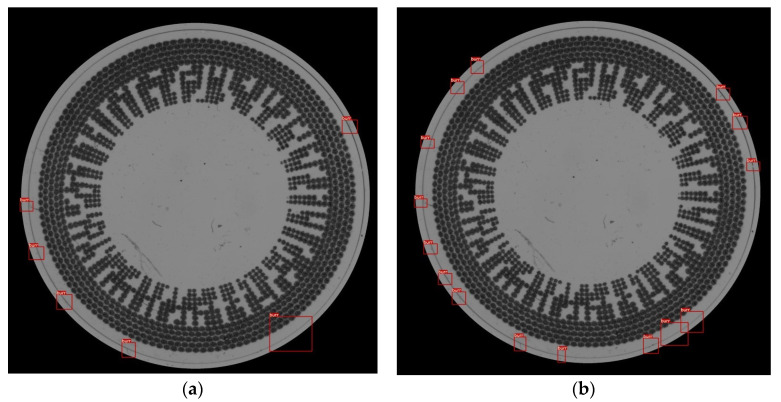
A comparison between the results of using or not using correction criterion function in the algorithm based on the area-based algorithm. (**a**) A result from the algorithm using window-sliding method; (**b**) A result from the algorithm without window-sliding method.

The window-sliding method is proposed with the objective of addressing the issue of uneven thickness around the entire circumference of the contact lens. The root cause of this uneven thickness on the contact lens rim stems from the imperfect placement of the contact lens in the contact lens holder. Tilt angles during its placement can lead to the conditions observed in the captured images as shown in Figure 7. Therefore, the window-sliding method is proposed to reevaluate the criteria defined by Equations (7) and (8) by calculating the average thickness within a specified local region of interest. This calculation is performed iteratively along the length of the circular rim. If the CLensRimVision algorithm is executed without the window-sliding method, the criteria defined by Equations (7) and (8) are calculated using the average value of the entire input of the contact lens. We conducted a comparative test between the results of using the window-sliding technique and tnot using it, as presented in Figure 13a,b, respectively. The testing results are based on the area-based approach, with both results having an extended and shrink area ratio set to 0.3 and a split image size of 10 pixels. As a result, the image in Figure 13a can detect fewer defects than the image in Figure 13b. Most of the detections in Figure 13b do not represent actual defects; instead, they are normal curves without defects. However, their values may fall outside the acceptable range during the defect detection stage. We can conclude that Figure 13a yields more precise results in defect detection in this case, aided by the window-sliding technique.

### 6.2. Effectiveness of Correction of Criteria Function

**Figure 14:**
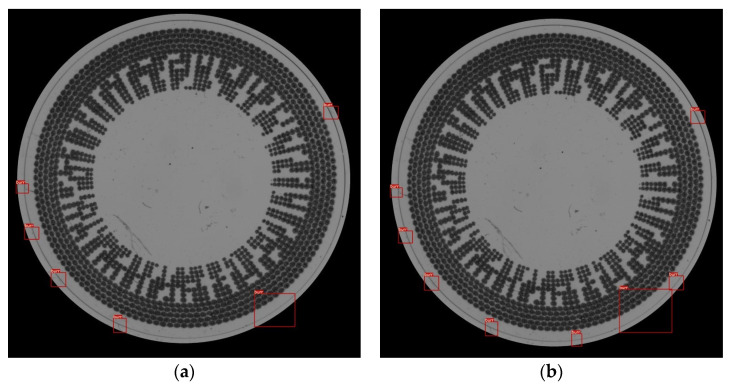
A comparison between the results of using or not using correction criterion function in the algorithm. (**a**) A result from the algorithm using correction of criterion; (**b**) A result from the algorithm without correction of criterion.

The correction of the criterion function is used to eliminate spike values from the local regions of interest in the window-sliding method. These spikes can potentially occur due to defects in the contact lens. However, when finding the average value of the local area, we do not want to include these spikes, as they may lead to an inaccurate average value for either the thickness (in the case of the thickness-based approach) or area (in the case of the area-based approach) within the local region. The purpose of this function is to remove outliers from the average criterion value in the local region that could interfere with the overall calculated average value. The correction of the criterion function employs statistical mathematics to limit the range of the average local value, as defined in Equation (6), in which we manually set the proportion as factor F. Assuming that there are extended spikes resulting from defects, mathematically, the average will be higher than in cases without any defects. Conversely, if a local region has cuts or shrinkage defects in a linear shape within the region of interest, the average value will be lower than usual. In Figure 14a, there is a demonstration of a contact lens image that has been tested for defect detection using the CLensRimVision algorithm with the correction criteria function. In contrast, Figure 14b shows the result without using the correction criteria function. The results reveal that the example image using the correction criteria has fewer detections compared to the other image without the correction criteria. This is because it detects only the defects that appear on the rim of the contact lens. Figure 14b has some detections that are false positives for defects because they may have higher or lower average values than they should. Consequently, some values that should not be detected are also identified as defects on the rim.

### 6.3. Characteristics of Criterion-Based Methods

In the CLenRimVision algorithm, there are two criterion-based methods available: the thickness-based and the area-based approaches. Both methods serve the same purpose, which is to count the number of black pixels within the region of interest during the window-sliding process. In this section, we aim to highlight the characteristics of each approach, as they may be suitable for different applications and objectives. Each approach comes with its own set of required parameters for the shrink value and extended value used in the calculation of the acceptable range. These parameters offer the flexibility to meet the standards and specific industry needs associated with different contact lens grades, such as economy-priced beauty contact lenses, premium contact lenses, and myopia contact lenses. 

When compared to physical contact lenses, the thickness-based method is more intuitive to configure within the algorithm. This is because the pixel thickness can be easily converted into the physical length, thanks to standardized quality control processes. In the premium contact lens industry, the thickness-based method has proven to be effective in quality control, as it uses micron units to precisely identify the required thickness adjustments, whether for shrinking or extending. Therefore, it can be concluded that the thickness-based method is more straightforward and suitable for most cases. 

Conversely, the area-based approach may not exhibit a direct correlation with physical contact lenses. This approach relies heavily on sensitivity when configuring parameters, especially when setting the window size in the window-sliding process. This sensitivity arises from the fact that the area of black pixels in the counting method is relatively proportional to the size of the split image in the window-sliding technique. To illustrate, if the area of the split image changes from 10 to 60 pixels while assuming a constant thickness, the area difference between a 10-pixel split image and a 60-pixel split image would be six-fold. As mentioned earlier, this highlights the importance of a deep understanding of the algorithm when using the area-based method. However, it is worth noting that, in certain scenarios, the area-based method can deliver slightly superior results compared to the other approach.

### 6.4. Optimal Value of Pixels Size of Spilt Image

According to the results presented in Table 2, it is evident that the overall performance is significantly better with a split image pixel size of 60 pixels compared to that of 10 pixels. This leads to the conclusion that the 10-pixel size is limited in its ability to detect larger defects, resulting in a lower accuracy compared to that of the 60-pixel size. To conclude, our experiments have demonstrated that the optimal choice for the pixel size of split images is 60 pixels. This selection has been substantiated by the fact that it can effectively encompass defects of varying sizes. On the other hand, smaller sizes, such as a size of 10 pixels, do not enhance the accuracy since they are incapable of detecting larger burrs.

## 7. Conclusions

This research aims to provide a comprehensive understanding of the intricate technique required to detect burr defects on contact lenses. These defects may manifest as variations in thickness along the rim circle of the contact lens. Building upon these observations and our research findings, we introduce CLensRimVision, an algorithm specifically designed for detecting defects on the rims of contact lenses—an essential part that is in contact with the human eye. The proposed algorithm consists of three main components: image processing, circle detection, and defect detection. In defect detection, we developed a thoughtful window-sliding technique that overcomes the limitations posed by having an uneven thickness. Additionally, a correction criteria method was employed to reevaluate the correct average value, which is crucial for determining the acceptable range. This range calculation involves variables for shrinkage and extension, making the algorithm adaptable to various parameter inputs. Furthermore, we introduced two criteria for different detection purposes: the area-based and thickness-based approaches. As a result, we have generated prediction accuracy results based on different parameter settings and the use of these two criteria. The results of the contact lens defect detection algorithms under different parameter settings are shown, showcasing their performance in terms of precision, recall, and average precision (AP of 0.5). Notably, the “thickness-based” algorithm consistently demonstrates competitive levels of precision and recall across various settings. The choice of the shrink/extended thickness criterion significantly influences its performance, with lower criteria favoring recall. Additionally, a greater factor correction of the criterion contributes to a slightly better precision. The algorithm performs optimally with a pixel size of split images set to 20, consistently achieving a high AP of 0.5. On the other hand, the “area-based” algorithm has proven to be competitive, particularly when the shrink/extended criterion is set to 0.4 or 0.5. It offers a balanced trade-off between precision and recall, with a significantly better performance achieved with a higher size of window. Both algorithms exhibit sensitivity to parameter settings, which emphasizes the need to tailor the choice of algorithm to specific application requirements and consider the trade-off between precision and recall. 

Additionally, we engage in a discussion on the proposed technique, which helps mitigate the diversity in rim thickness that can lead to errors and outliers, affecting the accuracy of the average value in each window sliding. This discussion aims to establish the meaningful and beneficial integration of each step within the algorithm. We believe that our work holds the potential to benefit the contact lens industry, offering applications in production or serving as a foundation for further implementations.

For our future work, several possibilities exist for extending this research or developing new algorithms capable of detecting other types of defects, such as hair and foreign particles. However, these defect types may not necessarily be related solely to the rim of the contact lens and may require further exploration and new techniques.

## Figures and Tables

**Table 1:** Parameters in offline process.

Sections	Parameter Name	Domain	Default
Image processing	H color space range	0 to 180	0 to 180
	S color space range	0 to 255	0 to 18
	V color space range	0 to 255	197 to 255
	Morphological Dilate	0 to 30	5
	Morphological Erode	0 to 30	5
Circle detection	Size of radius circle in range	integer	1200 to 1350
	Center circle position	(x, y) in integer	(2010,1050)
	Max area of a contour	integer	50,000
Criterion	Size of separated image	0 to 200	10
	Number of images above	15 to 100	50
	Number of images below	15 to 100	50
	Extended thickness pixel criterion (thickness based)	0 to 30	>3
	Shrink thickness pixel criterion (thickness based)	0 to 30	>3
	Extended area ratio criterion (area based)	0 to 3.0	>0.1
	Shrink area ratio criterion (area based)	0 to 3.0	>0.1
	Factor of SD to average	0 to 3.0	0.5

**Table 2:** Evaluation result with test set images for image processing algorithm.

Algorithm	Shrink/Extended Criterion	Number of Images Above/Below	Pixels Size of Spilt Image	Factor Correction of Criterion	Precision	Recall	AP@0.5
Thickness based	6	20	10	0.5	0.870	0.955	0.913
	8	20	10	0.5	0.918	0.924	
	10	20	10	0.5	0.951	0.911	
Thickness based	6	20	60	0.5	0.888	0.955	0.924
	8	20	60	0.5	0.925	0.940	
	10	20	60	0.5	0.959	0.911	
Area based	0.3	20	10	0.5	0.711	0.980	0.809
	0.4	20	10	0.5	0.821	0.971	
	0.5	20	10	0.5	0.894	0.955	
Area based	0.3	20	60	0.5	0.880	0.971	0.937
	0.4	20	60	0.5	0.951	0.955	
	0.5	20	60	0.5	0.980	0.940	

Setups of the value of shrink and extended criterion are identical, which is called the symmetric setting criterion. Similarly, symmetric setting is when the number of images above and below are identical.

## Data Availability

Data is contained within the article.

## References

[1] Lovrec-Krstič T., Orthaber K., Maver U., Sarenac T. (2023). Review of Potential Drug-Eluting Contact Lens Technologies. Materials.

[2] Ţălu Ş., Ţǎlu M., Giovanzana S., Shah R. (2011). A Brief History of Contact Lenses. Hum. Vet. Med..

[3] Harris V., Pifer R., Shannon P., Crary M. (2023). Comparative Evaluation of *Pseudomonas aeruginosa* Adhesion to a Poly-(2-Methacryloyloxyethyl Phosphorylcholine)-Modified Silicone Hydrogel Contact Lens. Vision.

[4] Lee M.-J., Park S.-Y., Sung A.-Y. (2022). Ophthalmic Hydrogel Contact Lens Material Containing Magnesium Oxide Nanoparticles and 3-(Trifluoromethyl)styrene for Biomedical Application. Micromachines.

[5] Meretoudi A., Banti C.N., Raptis P.K., Papachristodoulou C., Kourkoumelis N., Ikiades A.A., Zoumpoulakis P., Mavromoustakos T., Hadjikakou S.K. (2021). Silver Nanoparticles from Oregano Leaves’ Extracts as Antimicrobial Components for Non-Infected Hydrogel Contact Lenses. Int. J. Mol. Sci..

[6] García-Marqués J.V., Talens-Estarelles C., García-Lázaro S., Cerviño A. (2022). The Effects of Soft Contact Lens Wear on The Tear Film and Meibomian Gland Drop-Out and Visibility. Life.

[7] Seggio M., Nostro A., Ginestra G., Quaglia F., Sortino S. (2019). Contact Lenses Delivering Nitric Oxide under Daylight for Reduction of Bacterial Contamination. Int. J. Mol. Sci..

[8] Seibel E.J., Trilsch W.R., Lee D. (1988). Evaluating Soft Contact Lens Quality: A Manufacturer's Perspective. Am. J. Optom. Physiol. Opt..

[9] Sohal A.S. (1988). Quality Control in Soft Contact Lens Manufacture—A Case Study. Int. J. Qual. Reliab. Manag..

[10] Torres M., Santhanam K.S.V. (2014). Quality Control of Silicone Hydrogel Contact Lenses by Impedance Spectroscopy. Mater. Res. Soc. Symp. Proc..

[11] Mana N., Lim C., Chong Y.F., Yazid H., Mohd Ali Y. (2023). A Review on Contact Lens Inspection. Indones. J. Electr. Eng. Comput. Sci..

[12] Kim G.-n., Kim S.-h., Joo I., Kim G.-b., Yoo K.-h. (2023). Center Deviation Measurement of Color Contact Lenses Based on a Deep Learning Model and Hough Circle Transform. Sensors.

[13] Luo K., Kong X., Zhang J., Hu J., Li J., Tang H. (2023). Computer Vision-Based Bridge Inspection and Monitoring: A Review. Sensors.

[14] Smith A.D., Du S., Kurien A. (2023). Vision Transformers for Anomaly Detection and Localisation in Leather Surface Defect Classification Based on Low-Resolution Images and a Small Dataset. Appl. Sci..

[15] Chung S.-T., Hwang W.-J., Tai T.-M. (2023). Keypoint-Based Automated Component Placement Inspection for Printed Circuit Boards. Appl. Sci..

[16] Rudenko M., Plugatar Y., Korzin V., Kazak A., Gallini N., Gorbunova N. (2023). The Use of Computer Vision to Improve the Affinity of Rootstock-Graft Combinations and Identify Diseases of Grape Seedlings. Inventions.

[17] Gao Z., Qiu Y., Zhou J. (2022). Paper Defect Detection Algorithm Based on Mathematical Morphology and Computer Vision. J. Phys. Conf. Ser..

[18] Zuo D., Hu H., Qian R., Liu Z. (2017). An Insulator Defect Detection Algorithm Based on Computer Vision. Proceedings of the 2017 IEEE International Conference on Information and Automation (ICIA).

[19] Rebsamen M., Boucheix J.-M., Fayol M. (2010). Quality Control in the Optical Industry: From a Work Analysis of Lens Inspection to a Training Programme, an Experimental Case Study. Appl. Ergon..

[20] Kuo C.-F.J., Lo W.-C., Huang Y.-R., Tsai H.-Y., Lee C.-L., Wu H.-C. (2017). Automated Defect Inspection System for CMOS Image Sensor with Micro Multi-Layer Non-Spherical Lens Module. J. Manuf. Syst..

[21] Chang C.-L., Wu W.-H., Hwang C.-C. (2015). Automatic Optical Inspection Method for Soft Contact Lenses. Proceedings of the International Conference on Optical and Photonic Engineering (icOPEN 2015).

[22] Burdescu D.D., Brezovan M., Ganea E., Stanescu L., Blanc-Talon J., Philips W., Popescu D., Scheunders P. (2009). A New Method for Segmentation of Images Represented in a HSV Color Space. Advanced Concepts for Intelligent Vision Systems.

[23] Sural S., Qian G., Pramanik S. Segmentation and Histogram Generation Using the HSV Color Space for Image Retrieval. Proceedings of the International Conference on Image Processing.

[24] Bora D.J., Gupta A.K., Khan F.A. (2015). Comparing the Performance of LAB* and HSV Color Spaces with Respect to Color Image Segmentation. Int. J. Emerg. Technol. Adv. Eng..

[25] Marques O. (2011). Morphological Image Processing. Practical Image and Video Processing Using MATLAB.

[26] Hasan S.M.A., Ko K. (2016). Depth Edge Detection by Image-Based Smoothing and Morphological Operations. J. Comput. Des. Eng..

[27] OpenCV Geometric Transformations of Images. https://docs.opencv.org/3.4/da/d6e/tutorial_py_geometric_transformations.html.

[28] Puhan N.B., Sudha N., Hegde A.S. A New Iris Liveness Detection Method against Contact Lens Spoofing. Proceedings of the 2011 IEEE 15th International Symposium on Consumer Electronics (ISCE).

[29] Ramlee I., Ibrahim S., Leow W.Z., Yusoff M. (2020). A Review of Detecting Outliers in a Circular Regression Model. IOP Conf. Ser. Mater. Sci. Eng..

[30] Ou Y., Deng H., Liu Y., Zhang Z., Ruan X., Xu Q., Peng C. (2022). A Fast Circle Detection Algorithm Based on Information Compression. Sensors.

[31] Jin Y., Wang Z., Zhu L., Yang J. (2011). Research on In-Line Glass Defect Inspection Technology Based on Dual CCFL. Procedia Eng..

[32] Huang Y. (2020). A Novel Direct Structured-Light Inspection Technique for Contaminant and Defect Detection. arXiv.

[33] Cicconet M., Geiger D., Werman M. (2015). Complex-Valued Hough Transforms for Circles. arXiv.

[34] Smith J. Central Limit Theorem (CLT): Definition and Key Characteristics. Financial Analysis. https://www.investopedia.com/terms/c/central_limit_theorem.asp.

